# Adaptation of Human Influenza Viruses to Swine

**DOI:** 10.3389/fvets.2018.00347

**Published:** 2019-01-22

**Authors:** Daniela S. Rajao, Amy L. Vincent, Daniel R. Perez

**Affiliations:** ^1^Department of Population Health, University of Georgia, Athens, GA, United States; ^2^Virus and Prion Research Unit, USDA-ARS, National Animal Disease Center, Ames, IA, United States

**Keywords:** influenza A virus, swine, human, interspecies, adaptation, host range

## Abstract

A large diversity of influenza A viruses (IAV) within the H1N1/N2 and H3N2 subtypes circulates in pigs globally, with different lineages predominating in specific regions of the globe. A common characteristic of the ecology of IAV in swine in different regions is the periodic spillover of human seasonal viruses. Such human viruses resulted in sustained transmission in swine in several countries, leading to the establishment of novel IAV lineages in the swine host and contributing to the genetic and antigenic diversity of influenza observed in pigs. In this review we discuss the frequent occurrence of reverse-zoonosis of IAV from humans to pigs that have contributed to the global viral diversity in swine in a continuous manner, describe host-range factors that may be related to the adaptation of these human-origin viruses to pigs, and how these events could affect the swine industry.

## Introduction

Influenza is one of the most devastating respiratory pathogens of pigs and humans and continues to threat animal and public health with the continuing possibility of outbreaks or a pandemic. The intricacies of influenza A viruses (IAV) at the human-swine interface dates back to the 1918 pandemic. For several decades, it was hypothesized that pigs played a role in the origin of the 1918 H1N1 pandemic virus (1). Although there is evidence suggesting that the pandemic virus did not originate from pigs and that the classical swine H1N1 virus was in fact derived from the 1918 human virus (2), the bias perceiving swine as the source of IAV to humans still remains.

The ecology of IAV is complex and involves a broad range of avian and mammalian host species. IAVs are enveloped, segmented RNA viruses in the family *Orthomyxoviridae* (3). The virus genome is composed of eight negative-sense, single-stranded viral RNA (vRNA) segments that encode between 10 and 17 viral proteins depending on the strain (4–6). Each RNA segment forms the viral ribonucleoprotein complexes (vRNPs) with the nucleoprotein (NP) and the three polymerase proteins (PB2, PB1, and PA). Two major glycoproteins are projected on the virus envelope, hemagglutinin (HA), and neuraminidase (NA) (7). Based on the antigenic properties of the HA and NA, IAV are divided into 18 HA subtypes (H1–H18) and 11 NA subtypes (N1–N11) (7–9).

Influenza viruses have high mutation rates and are constantly changing, which enables the virus to quickly adapt to changes in the host environment, as is the case during interspecies transmission. The rapid evolution results from two mechanisms: reassortment and point mutations (10). Reassortment occurs when two different strains infect the same cell of a given host, allowing for exchange of intact gene segments. When reassortment involves either the HA or NA segments, it is termed antigenic shift. Point mutations occur due to an error prone polymerase devoid of a proof-reading and correction mechanism. When point mutations are fixed in the HA or NA segments, usually a result of escape from immune pressure, it is termed antigenic drift. Both of these mechanisms play pivotal roles in the emergence of novel influenza viruses that could jump the host barrier. Once the virus jumps into a new host, it must adapt and change to be able to spread and become established in the new population.

In this review, we describe the role of pigs in the interspecies transmission of influenza and how their susceptibility to different viruses can affect the overall epidemiology of swine influenza. We discuss the factors that have been implicated in the interspecies transmission of influenza with an emphasis on the human-swine interface. We then provide an overview of human-to-swine IAV spillover events that significantly affected the epidemiology of viruses circulating in swine and how these viruses can have a negative effect on the control of influenza in pigs.

## Why Pigs Become Infected With Viruses From Other Species?

To result in a successful replicative cycle, influenza viruses must efficiently infect the host cell, replicate, and produce functional virus progeny that will be released and infect new cells. The first step for infection is the attachment of the HA protein to the cell receptor. The HA is a type I transmembrane glycoprotein, present as a homotrimer on the virus' surface, each monomer carrying a transmembrane anchor and a small cytoplasmic tail. The proteolytic cleavage of the precursor HA0 produces two subunits, HA1 (globular head) and HA2 (stem). The receptor binding site (RBS) forms a shallow pocket at the distal tip of the HA1 head and consists of a base of four highly conserved amino acid residues (Y98, W153, H183, and Y195, numbering based on the H3 subtype) that are bordered by the 130-loop, the 190-helix and the 220-loop (11–13).

**Figure 1:**
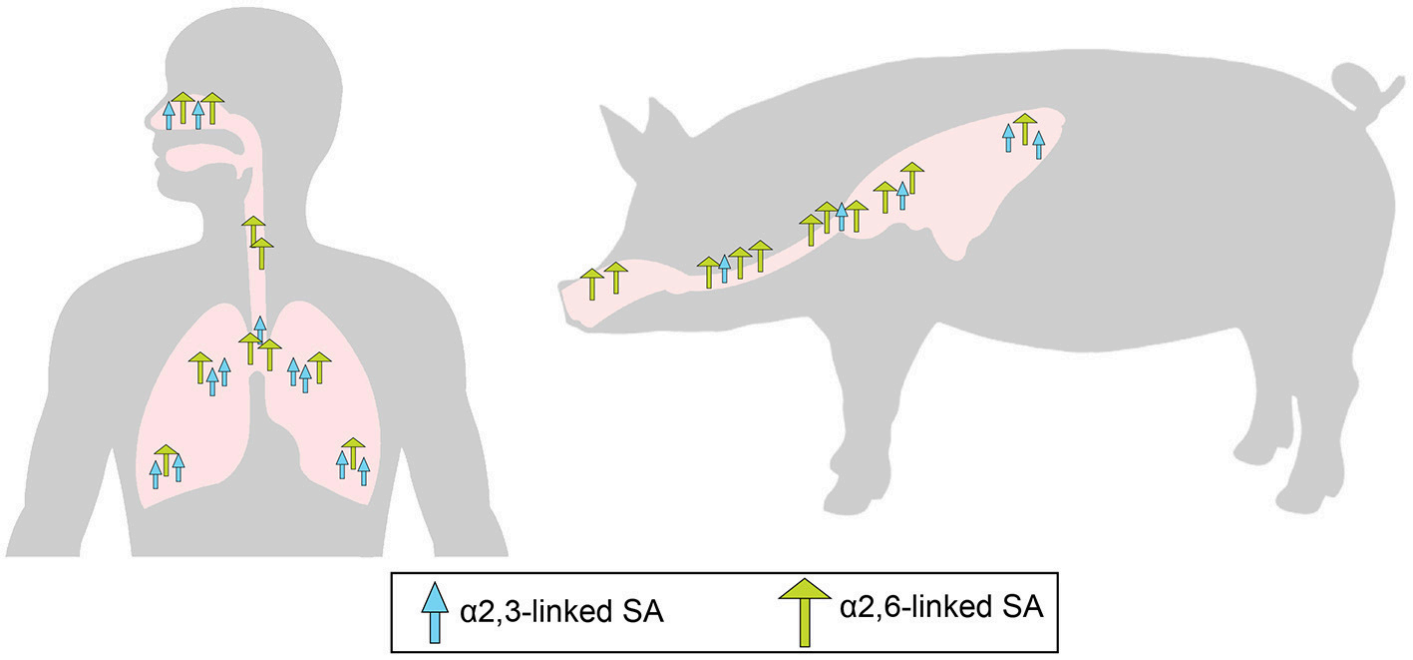
Overall distribution of α2,6-linked sialic acid (SA; green long arrow) and α2,3-linked SA (blue short arrow) in the epithelium of the respiratory tract of pigs (18, 19) and humans (14, 15). Adapted from de Graaf and Fouchier (20).

Through the RBS, influenza viruses bind to terminal sialic acid (SA, N-acetylneuraminic acid) moieties in glycoprotein or glycolipid receptors on the host cell surface. The SAs are usually bound to the penultimate galactose (Gal) in two major conformations: α2,3SA or α2,6SA (13). Differences in the type of SA linkage found in receptors expressed in different host species have a major impact on the host restriction of IAVs. Sialic acids with α2,3-linkage are predominantly expressed on epithelial cells in the intestinal and respiratory tracts of birds while the epithelial cells in the upper respiratory tract of humans contains predominantly α2,6-linked SA receptors (14–17) (Figure 1). Most avian influenza viruses preferentially bind to α2,3-SA, whereas human and other mammalian influenza viruses preferentially recognize α2,6- SA receptors (21–23).

Pigs have been historically believed to be intermediary hosts, or “mixing vessels,” of influenza viruses due to their susceptibility to infection with both human-origin and avian-origin IAV and their propensity for the generation of reassortant viruses (24–27). Pigs have a similar distribution to humans of α2,3-SA and α2,6- SA receptors in the respiratory tract (Figure 1). As in humans, α2,6-linked SA receptors predominate in the upper respiratory tract of pigs, but α2,3-SA receptors are present in low quantities in swine tracheas, and the frequency increases toward the lower respiratory tract (18, 19) (Figure 1). The presence of both types of SA receptors in swine airways supports the potential role of pigs as “mixing vessels.” However, such distribution of α2,3- and α2,6-SA receptors is similar in swine and humans (15, 18), and it must be noted that avian viruses do not usually transmit from pig-to-pig as is also the case in humans (28, 29). Humans can also become infected with avian-origin IAVs directly from avian sources and could potentially provide the environment for the adaptation of avian viruses (30–32). Hence, generation of reassortant viruses with pandemic potential may not require swine as intermediate hosts. However, as highlighted by the 2009 pandemic (33), while swine are not required, they may serve as intermediate hosts for generation of reassortant viruses with the ability to cause human pandemics. The 2009 pandemic has led to an increased concern about the transmission of swine viruses to humans. However, improved surveillance of swine IAV after the pandemic has shown that human viruses are transmitted to pigs, and have resulted in sustained onward transmission, far more frequently than swine viruses have infected humans (34). This lower host barrier observed for human viruses in pigs can be explained in part by the similar receptor distribution in both species and the shared preference for α2,6-linked SA receptors between human and swine viruses (22, 26).

## What Are The Mechanisms For Adaptation of Human Influenza Viruses To Pigs?

Although IAV transmission events from humans to pigs are continually detected globally and despite the similarities of receptor preference and distribution between the two species, whole human IAV rarely become established in swine. Typically, these viruses reassort and emerge with only some of the human-origin viral gene segments persisting, often with marked genetic differences from the precursor strain (34–36). This implies that adaptation factors other than the receptor linkage-type specificity are required for human-origin viruses to be transmitted and subsequently become endemic in swine populations.

The adaptation of influenza viruses between humans and pigs is likely driven by selective pressures or bottlenecks imposed to the virus population during IAV host jump, as a result of the changes in the host environment (37, 38). Several factors may affect these selective pressures during interspecies transmission, either within the virus or the host. Receptor-binding specificity and affinity, balance between HA and NA content, temperature of the host, and host-specific immune factors may be some of these factors. However, the differences in the selective pressure between humans and swine and how they may differently affect virus adaptation are not entirely understood, and some of the currently know differences are discussed below.

### Binding Determinants of Host Range

**Figure 2:**
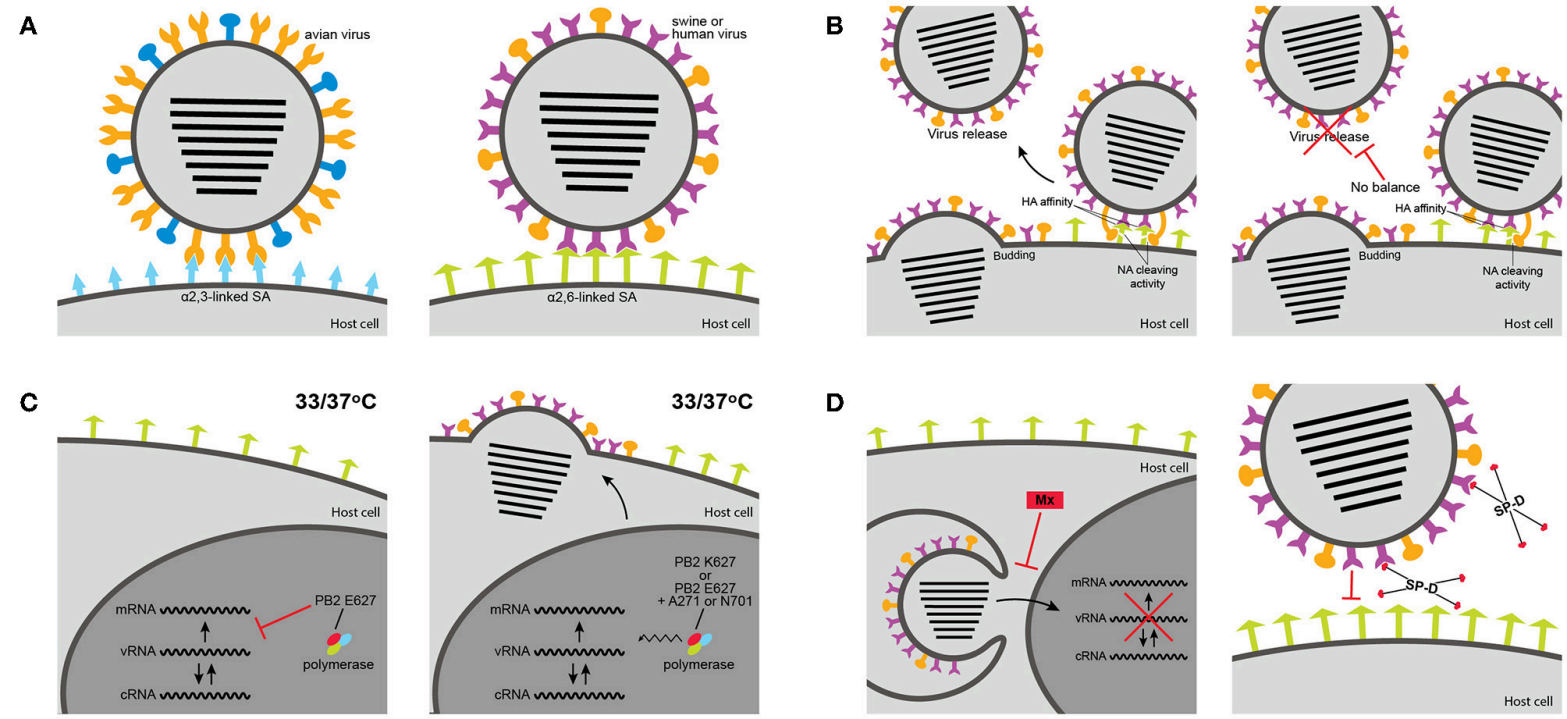
Host range determinants of influenza A viruses (IAV). **(A)** Avian influenza virus HA protein recognize short α2,3-linked sialic acid (blue), whereas HA from human and swine IAV recognize long α2,6-linked sialic acid (green). **(B)** The balance between the HA binding affinity and the NA activity to cleave sialic acid receptors is important for replication and adaptation to a new species. If a virus has strong biding affinity but low cleavage activity replication may be reduced. **(C)** The PB2 polymerase has an impact in the optimal replication temperature of IAV and can restrict host range. K627 increases replication at the low temperature of human or swine upper airway. E627 decreases replication at low temperatures, unless in combination with A271 or N701. **(D)** The sensitivity of a virus to host-specific innate immune factors can restrict interspecies transmission of IAV. To be able to replicate and spread in a new host, IAV must become resistant to the antiviral activity of interferon-induced Mx protein or to the neutralizing activity of surfactant protein D (SP-D) from that particular host. Adapted from Cauldwell et al. (39).

**Figure 3:**
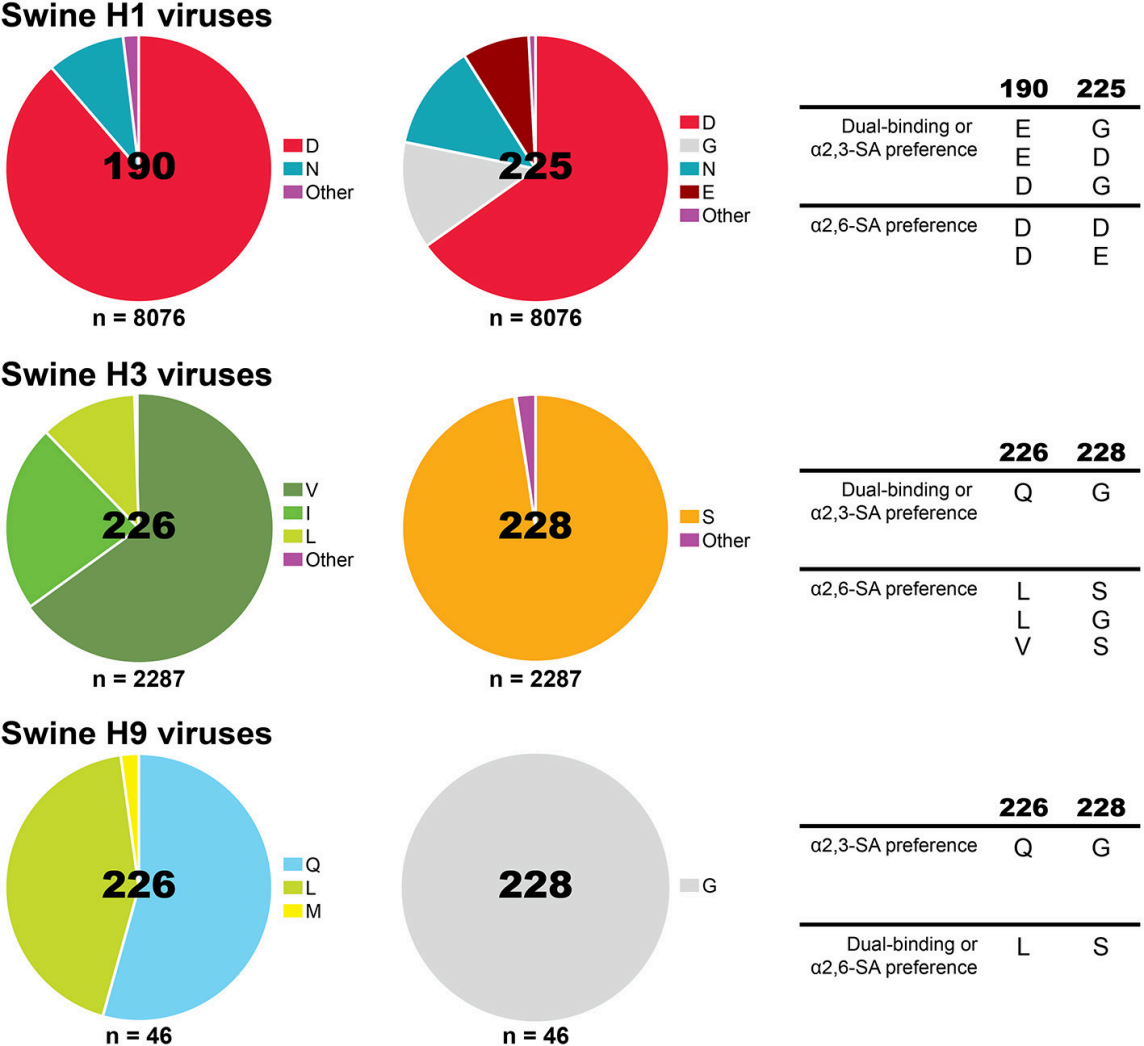
Proportion of amino acids found in influenza A viruses circulating in pigs globally at the HA receptor-binding site positions previously shown to impact receptor-specificity for H1, H3, and H9. Analysis was performed using the Influenza Research Database Sequence Variation (SNP) tool (44). Sequences with 100% identity were removed resulting in a set of 8076 H1 HA, 2287 H3 HA, and 46 H9 HA swine IAV sequences. The amino acids previously shown to change receptor-binding specificity are displayed on the right.

Specific amino acid residues at the influenza HA are required for binding to either α2,3-SA or α2,6-SA receptors and specific amino acid substitutions at the RBS of the HA can alter receptor-binding specificity and facilitate host jump (Figure 2A). In H1 subtype viruses, positions 190 and 225 were shown to have an impact in receptor specificity. The combination of E190/G225, E190/D225, or D190/G225 in the RBS of the HA, found in avian viruses and late stage 2009 pandemic H1N1 strains, results in dual receptor-binding specificity, whereas D190/D225 and D190/E225, combinations found in seasonal human viruses, results in human-type receptor specificity (40–42). As for H3 and H9 viruses, positions 226 and 228 in the HA are critical for receptor specificity. Avian-adapted viruses usually present Q226/G228 and show dual-binding or α2,3-SA preference, but amino acid substitutions Q226L/G228S leads to receptor specificity switch to human-type receptor preference and is, therefore, more commonly found in human viruses (22, 43). Analysis of H1, H3, and H9 virus sequences from swine using the Influenza Research Database (44) revealed that swine viruses have mostly D190/D225 in H1 viruses, a fairly equal distribution between Q226/G228 and L226/G228 in H9 viruses, and the unique combination of amino acids in H3 viruses V226/S228 (Figure 3).

Receptor-binding specificity of influenza HA is not only mediated by changes in the sialic acid linkage, the structural length and topology of the glycans can also determine the binding specificity and affinity of IAV. Avian viruses were shown to bind to α2,3-linked SA carrying a shorter carbohydrate chain whereas human viruses bind preferentially to long α2,6-linked SA (45, 46). Moreover, avian HA binds to narrow α2,3-SA in a “cone-like” topology and human HA binds to long α2,6-SA in an “umbrella-like” topology, which are predominantly expressed in the human upper respiratory tissues (47). In general, human and swine viruses have been shown to recognize similar glycan structures on glycan microarrays, mainly branched α2,6-SA (48, 49).

### NA and M as Determinants of Host-Range

While the HA is involved with binding to SA receptors, the NA cleaves α2-3 and α2-6-linked SA residues from cellular surfaces and mucus through its sialidase enzymatic activity and mediates the release of newly synthesized viruses from the host cells (7). For an optimal viral replication, balanced activities between the HA binding affinity and the NA enzymatic function are expected. The ideal HA-NA balance seems to be an important factor in host adaptation (Figure 2B). The HA-NA balance was shown to be crucial for the adaptation of the 2009 pandemic H1N1 virus to humans, since balanced HA and NA activities were seen in the human strains but not in precursor swine viruses (50) and this balance resulted in increased replication and transmissibility in ferrets (51). Additionally, adaptation of H5 and H7 viruses from wild birds to chickens led to selective changes in both HA and NA, maintaining a balance between binding and cleavage that was important for replication and transmission in the new host (52). These chicken-adapted H5 and H7 viruses possess a shorter NA due to the deletion of several residues in the stalk domain that were shown to enhance replication and virulence in chickens but block respiratory transmission in ferrets (53, 54).

In addition to the NA, the matrix (M) gene segment has been shown to be a critical determinant of respiratory transmission efficiency of IAV in new hosts. The M segment was implicated with the increased transmissibility of the 2009 pandemic H1N1 virus in animal models (55, 56), suggesting it played an important role on the spread of the virus in humans. In pigs, the combination of the NA and M genes from the 2009 pandemic virus was essential to facilitate efficient replication and transmissibility (57). Interestingly, reassortant H1 and H3 swine-origin viruses containing the M gene of the 2009 pandemic virus have caused almost yearly zoonotic outbreaks in humans, more frequently than was observed prior to the pandemic, confirming that the M gene plays a role in adaptation and transmission of swine viruses in humans (58–60).

### Temperature Determinants of Host-Range

The virus polymerase (comprised of viral proteins PB1, PB2, and PA) was also shown to be a major determinant for host range of influenza viruses (61) (Figure 2C). This host restriction has been attributed to a single residue in the PB2 gene, amino acid 627, and is largely associated with the optimal temperature of replication of IAVs (62, 63). While the human upper respiratory tract temperature is around 33°C, in the avian intestinal tract the temperature is closer to 41°C. Therefore, enhanced replication at lower temperature should correlate with enhanced replication in the upper airway of humans and consequently improve transmission. Lysine (K) at position 627 in PB2, present in the vast majority of human viral isolates (64), was correlated with increased polymerase activity, virus replication and transmission in mammals (65–67), including enhanced replication of an avian virus in pigs (68). Replication and polymerase activity of different avian viruses, which predominantly possess glutamic acid (E) at position 627, were reduced at low temperature in mammalian cells (65, 66, 69).

The temperature of the upper respiratory tract of pigs is approximately 37°C and higher (approximately 39°C) in the lower respiratory tract. Interestingly, most swine isolates that have a PB2 of avian-origin retain the avian signature E627, including the predominant North American triple reassortant internal gene (TRIG) constellation viruses, the predominant Eurasian avian-like viruses, and even the 2009 pandemic H1N1 viruses (70, 71). The presence of the avian-like E627 in swine viruses usually does not result in the temperature sensitivity observed for avian viruses in mammalian cells (69), suggesting that these viruses can replicate at temperatures of avian intestines and human airways. Other residues, such as A271 and N701, were shown to compensate for the absence of K627 in these swine or swine-origin viruses and contribute to virus growth and transmission in swine and other mammalian species, including humans (71–74).

### Immune Determinants of Host-Range

Following influenza infection in respiratory epithelial cells, acute inflammation leads to activation of the innate immune response through pro-inflammatory cytokines or chemokines (75). Type-I interferons (IFN-α/β) are cytokines quickly secreted after IAV infection. Type-I IFN mediated responses to IAV results in the expression of several antiviral proteins (76, 77). The Mx proteins are a family of large GTPases that are central to the antiviral activity of IFN against IAV by blocking nuclear entry of the vRNPs (78, 79). Sensitivity to interferon-induced Mx varies among different IAV strains and represents a barrier against transmission of avian influenza viruses to mammals: avian isolates are more susceptible to the antiviral action of murine Mx and human MxA proteins than human viruses (80, 81). The Mx sensitivity was shown to be determined by a cluster of surface-exposed amino acids on the viral NP (81, 82). Interestingly, serial passage in mice of a virus that is sensitive to murine/human Mx activity leads to a single amino acid adaptive NP mutation that results in escape from the Mx activity, and the same mutation is also seen in human H7N9 isolates (83). Not surprisingly, some swine IAV strains with avian-origin NP tend to have a higher sensitivity to mouse Mx1 than human isolates (84). However, the 1918 pandemic H1N1 and the 2009 pandemic H1N1 viruses acquired resistance-associated substitutions on the NP protein that allow escape from human Mx (82). The functional Mx1 protein is expressed in the lungs of pigs experimentally infected with IAV (85). It seems that the precursor of the 2009 pandemic H1N1 virus acquired Mx-resistance mutations driven by the porcine Mx1 during its circulation in pigs prior to the pandemic, being able to partially resist the human MxA (82). The Eurasian avian-like viruses are similarly resistant to human MxA, however different mutations were attributed to this phenotype (86). It remains unknown whether human and swine viruses would have different sensitivities to the porcine Mx protein.

Surfactant protein D (SP-D) is a collectin of the innate immune system that also has early strong antiviral activity against IAVs. SP-D binds to carbohydrate moieties on the surface of influenza viruses (HA and/or NA), blocking attachment to epithelial cells and inducing phagocytic responses, resulting in non-specific virus neutralization and clearance (87). The susceptibility of different IAV to SP-D activity was shown to be dependent on the glycosylation pattern of the virus, particularly on the HA (88–90). Influenza strains of the H3 subtype tend to acquire and accumulate more glycosylations on the HA head as a mechanism to evade the antibody response in humans, but this in turn may make them more susceptible to the antiviral effect of SP-D. Interestingly, porcine SP-D has a higher affinity to bind IAV glycans than human or rat SP-D, resulting in stronger neutralization activity (91, 92). Therefore, differences in susceptibility to Mx or SP-D could be an important component in host restriction of influenza viruses that needs to be overcome, usually by changes in specific viral proteins, in order for a virus to adapt to a new species (Figure 2D).

The IAV NS1 protein plays an important role as an antagonist of the host IFN response by preventing the activation of retinoic acid-inducible gene 1 (RIG-I) or inhibiting processing of mRNA (93). Differences between the NS1 amino acid sequences may affect the functional IFN-antagonistic properties of the NS1 (94, 95). Consequently, NS1 and its ability to control IFN response could play a role in host range of IAV. Indeed, although the avian NS1 protein was able to control IFN-α/β response in human cells, the human type I IFN response appeared to limit the replication of the avian viruses, suggesting that the NS1 also contributes to the host specificity of IAV (96).

The adaptive immunity of an individual or population can also have a role in host range restriction of IAV. Even when a novel virus contains an ideal combination of factors that allow replication in the new host, as discussed above, previous cross-protective immunity might block even the initial infection. In some cases, the level of cross-protective immunity of the population may still allow infection but might block virus dissemination; however, naïve individuals will be at a higher risk for infection and may serve as sources of transmission. That was the case for the zoonotic infections with swine-origin viruses in recent years, in which the majority of affected individuals were children (58, 59). For these outbreaks, infection was observed in people with close contact with pigs, and transmission from human-to-human was rare, which was attributed to low levels of cross-protective immunity in the human population due to previous exposure to seasonal viruses (97). In pigs, however, there is a continuous introduction of naïve individuals and the majority of the population does not have previous immunity to viruses circulating in humans, increasing the chances of those viruses that have ability to infect pigs to become widespread.

## How Do Human Viruses Relate To The Evolution Of Swine Influenza Viruses?

**Figure 4:**
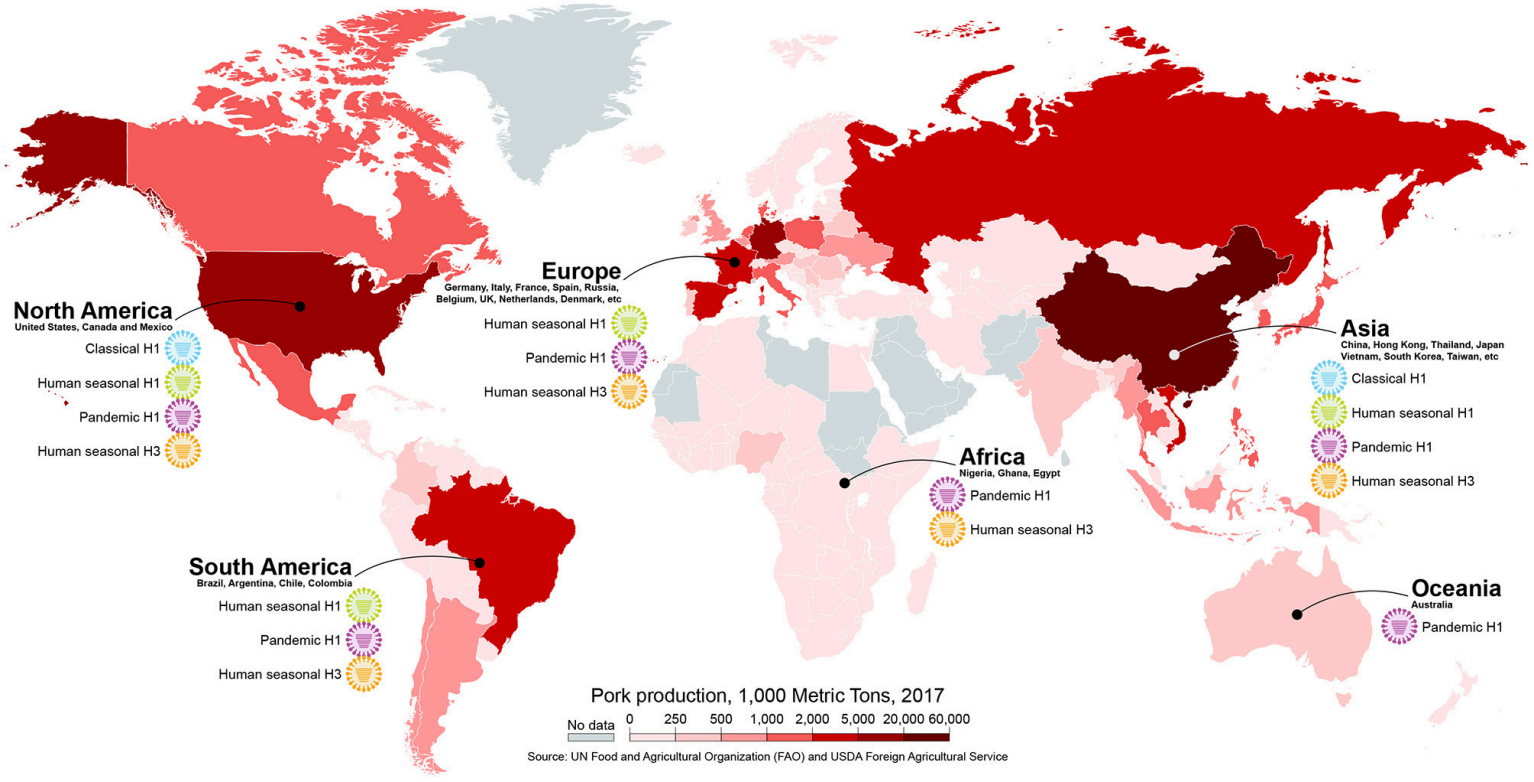
Different subtypes/lineages of human-origin influenza viruses circulating in swine in different continents. The map is colored according to pork production in 1,000 metric tons. Map created with mapchart.net.

Human-origin viruses have been repeatedly transmitted to swine worldwide and have had a major role on the epidemiology of swine IAV (34) (Figure 4). The classical swine H1N1 virus that emerged around the 1918 pandemic remained relatively antigenically stable for eight decades without causing major problems to swine producers. A novel triple-reassortant virus with human seasonal H3N2 surface genes emerged in the late 1990's in North America (27, 98) and led to reassortment with the classical viruses and subsequently gave rise to different antigenically distinct H3N2, H1N1, and H1N2 strains (99, 100). The triple-reassortant internal gene (TRIG) constellation, containing gene segments from a complex reassortment history among swine-, human- and avian-origin IAVs, became the predominant backbone of the viruses circulating in pigs in the U.S. (101, 102). Shortly after, two additional introductions of human-origin H1N1 resulted in the establishment of two new lineages of H1N1 and H1N2 viruses after reassortment with the TRIG strains, termed δ-lineages (103). After the spread in humans, the H1N1 pandemic 2009 virus (H1N1pdm) was quickly transmitted to swine in North America (104). And recently, a novel virus derived from 2010 to 2011 human seasonal H3 IAV led to establishment of a new H3-lineage that is genetically and antigenically distinct from previously circulating strains (105). The current scenario for the epidemiology of IAV circulating in North American swine consists of a highly diverse pool of viruses, with 14 phylogenetic clades of HA co-circulating (36, 101, 105, 106). It is clear how impactful the human-to-swine transmissions were to this current epidemiology: at least 10 of these phylogenetic clades have evolved from a human virus. If considering the hypothesis that the classical swine virus originated from the human 1918 pandemic virus, all of those clades should be considered of human-origin.

In Europe, a human-origin H3N2 virus descendent from the 1968 pandemic virus was introduced in the 1980's. This virus became widespread after reassorting with an avian-origin H1N1 virus that was introduced to European swine in 1979 and remains endemic to date (107, 108). Another human-origin virus, an H1N2, was detected in 1994, containing the H1 that evolved from a 1980 human seasonal H1N1 virus and a human-origin N2 that is distinct from the previously introduced H3N2 human-like virus. This virus acquired the internal gene constellation of the 1979 avian-like virus after reassortment and is now endemic in Europe (109, 110). As in the U.S., the H1N1pdm virus has been transmitted from humans to pigs in Europe establishing a new endemic lineage (108, 111). Recently, a triple-reassortant H3N2 virus with a human-origin HA from a 2004–2005 seasonal virus, N2 from endemic swine viruses, and the internal genes from H1N1pdm has spread in Denmark swine herds (112). In China and other countries in Asia, importation of live animals has resulted in the co-circulation of both European (or Eurasian) and North American TRIG virus lineages that contain human-origin genes (113–115). Additionally, reassortant genotypes between these lineages containing HA and/or NA genes from H1N1 and H3N2 human viruses have been detected in Asia since the 1960's and have become established in pigs (34, 116, 117).

Human-origin IAVs have been reported circulating in pigs in other countries where surveillance is limited (34, 118–120), including countries with large swine populations like Brazil (121, 122), Vietnam (123), Mexico and Chile (124). But, even in some of these cases where human-origin viruses or viral genes were reported in swine, it is not possible to infer if they have become endemic or predominant. However, in several cases, such as in Latin America, the human-origin swine viruses were most closely related to human seasonal strains that circulated many years earlier and were separated by long phylogenetic branches, suggesting that these viruses have circulated undetected in pigs for years prior to their recent detection (34, 118, 121, 124). Considering the frequency of human-to-swine transmissions in highly surveilled areas, it is likely that additional human-origin viruses have gone undetected in countries with low surveillance efforts.

In addition to the recurrent seasonal virus spillover events into swine populations, the H1N1pdm has been repeatedly transmitted from humans to swine globally (125). The H1N1pdm virus originated in Mexico from the reassortment between Eurasian and North American swine viruses and this novel virus may have circulated undetected for approximately 10 years before it gained the ability to infect humans (33). Soon after the initial spread of the H1N1pdm in the human population, the H1N1pdm virus was detected in pigs and since then transmitted from human to pigs throughout the world (104, 126–131). The virus has now become endemic in humans and circulates as a seasonal strain, increasing the possibility of spillovers to swine populations during influenza season each year. Owing to its swine-origin and yearly circulation in humans, continuous and frequent detection of the H1N1pdm virus in pigs has been reported globally (125, 132). The constant circulation and re-introduction of H1N1pdm globally has led to reassortment with endemic swine viruses and changed the genotypic characteristics of swine IAV by contributing several genes, most commonly the internal genes. In the U.S., the surface genes of the H1N1pdm are not frequently maintained, however most genotypes of H1 and H3 viruses contain at least one internal gene of pandemic lineage (133, 134). In Europe, the H1N1pdm virus has reassorted with endemic European viruses and gave rise to genotypes containing the internal genes from pandemic origin and some genotypes have maintained one or both surface genes of pandemic lineage (135). Interestingly, there is recent evidence of the independent antigenic evolution of the swine H1N1pdm virus in European pigs (136). In China, although Eurasian and North American viruses circulated prior to the 2009 pandemic without substantial evidence of reassortment, the introduction of the H1N1pdm led to the establishment of reassortant genotypes containing several internal genes from pandemic lineage (117). The H1N1pdm has been reported throughout the world in swine with frequent reassortment (137–139), even in countries that were previously considered influenza free like Australia and Norway (128, 140).

## How Do Human-Origin Viruses Affect Control Of Influenza In Swine?

The repeated transmission of human seasonal viruses to pigs has resulted in the establishment of several human-origin virus lineages globally, adding to the antigenic diversity of swine viruses. Global antigenic characterization has revealed that the antigenic diversity of H1 and H3 viruses circulating in pigs was largely a result of the frequent introductions of human-origin IAV into swine (35). These viruses then evolved antigenically, independent from human strains and often confined to their geographic areas, contributing to the overall global diversity, which consequently contributes to the challenges for effective vaccination programs in swine. Most vaccines used against influenza in swine are whole inactivated virus (WIV) vaccines combined with oil-in-water adjuvants typically given to sows to allow transfer of maternally derived antibodies to piglets (141). Recently, two novel platforms were licensed for use in pigs in the U.S. as alternatives to improve the efficacy of swine vaccines, a non-replicating alphavirus RNA vectored-vaccine and a live-attenuated influenza virus (LAIV) vaccine (142, 143).

Because most vaccines rely on the effective stimulation of the immune response against the surface HA glycoprotein, any changes that lead to antigenic drift, such as the incursion of novel human-origin viruses, can lead to vaccine mismatch. It was demonstrated that changes in only 6 amino acids in the HA account for major antigenic changes of swine H3 influenza viruses, and a single amino acid change can lead to significant antigenic drift (144, 145). Amino acids in similar positions at the HA were also associated with antigenic characteristics of H1 viruses (36). It is not surprising, therefore, that when novel human-origin viruses become established in pigs there are considerable antigenic differences from the circulating swine strains (105), and any vaccines available at the time are unlikely to provide immunity against these novel viruses. In addition to the lack of protection, vaccine mismatch can also have detrimental effects. When the vaccine stimulates a cross-reactive antibody response that fails to neutralize the virus, it can result in severe immune-mediated disease termed vaccine-associated enhanced respiratory disease (VAERD). Therefore, more effective vaccine technologies and vaccination strategies that improve the breadth of the immune response and avoid any negative effects are needed to increase protection against the antigenically diverse human-origin viruses that are continuously introduced in pigs.

## Concluding Remarks

Since the 2009 pandemic, renewed attention has been given to the interspecies transmission of influenza viruses between pigs and humans, bringing back the attention to the theory that pigs can serve as “mixing-vessels” of influenza viruses. However, it is not entirely clear if swine are in fact more susceptible to infection with avian viruses than humans. There is compelling evidence, though, that human viruses are frequently transmitted to pigs, and have had a significant impact on the diversity of viruses that circulate in pigs globally. Additional surveillance is necessary to understand the diversity of IAVs circulating in different regions and the participation of human-origin strains in this overall diversity. Surveillance is also critical for antigenic characterization of the strains that are circulating in a particular area to allow an accurate selection of representative vaccine strains that will provide an optimal protection. Moreover, despite the increasing evidence of the important role that human seasonal viruses have played in driving the genetic and antigenic diversity of IAV in swine, vaccine and sick leave policies for swine industry workers are not consistently employed but should be considered. Furthermore, understanding the mechanisms involved with host-range specificity and the adaptation to swine allows assessment of the risks posed by the introduction of novel viruses into the swine population, which is crucial for preparedness and to improve biosecurity measures to reduce the IAV burden to the swine industry.

## Author Contributions

DR, AV, and DP contributed to the conceptualization of the ideas, drafting and critical revision of the manuscript, and final approval. DR designed figures.

### Conflict of Interest Statement

The authors declare that the research was conducted in the absence of any commercial or financial relationships that could be construed as a potential conflict of interest.
